# Acute Kidney Injury Following Chimeric Antigen Receptor T-Cell Therapy: A Retrospective Cohort Analysis

**DOI:** 10.3390/jcm15218287

**Published:** 2026-10-24

**Authors:** Luc Delneste, Eddine Bendiab, Laura Platon, Caroline Bret, Charlotte Boullé, Nacim Benchabane, Jeremy Contier, Charles Herbaux, Patrice Ceballos, Kada Klouche

**Affiliations:** 1 Medical Intensive Care Unit, Montpellier University Hospital, 34090 Montpellier, France; 2 Department of Biological Hematology, Montpellier University Hospital, 34090 Montpellier, France; 3 Institute of Human Genetics, Unité Mixte de Recherche, Centre National de la Recherche Scientifique, University of Montpellier, 34396 Montpellier, France; 4 Department of Infectious and Tropical Diseases, Montpellier University Hospital, 34295 Montpellier, France; 5 TransVIHMI INSERM U1175 French National Research Institute for Sustainable Development (IRD) U233, University of Montpellier, 34394 Montpellier, France; 6 Clinical Hematology, Montpellier University Hospital, 34090 Montpellier, France; 7 PhyMedExp, School of Medicine, Inserm—French Institute of Health and Medical Research—CNRS (French National Centre for Scientific Research), University of Montpellier, 34295 Montpellier, France

**Keywords:** CAR-T cell, intensive care unit, acute kidney injury, CRS, ICANS

## Abstract

**Background**: Chimeric antigen receptor T-cell (CAR-T) therapy is associated with numerous adverse effects, among which acute kidney injury (AKI) remains poorly characterized. This study aimed to determine the incidence and risk factors of AKI following CAR-T therapy. **Methods**: This retrospective study was conducted among adult patients who received CAR-T cell therapy at an university hospital between April 2019 and December 2023. The incidence and risk factors for AKI, as well as its impact on overall survival and progression-free survival, were analyzed using univariate and multivariate analyses. **Results**: A total of 220 patients were included (mean age 63 ± 12 years), mostly treated for B-cell lymphomas. Pre-existing chronic kidney disease (CKD) was present in 26% of patients, and 24% had been admitted to the intensive care unit. The cumulative incidence of AKI was 20.5% (n = 45), occurring early after CAR-T infusion (4 days). Among AKI cases, 22% were KDIGO stage 3, and 15.6% required renal replacement therapy. In multivariate analysis, pre-existing CKD was strongly associated with AKI occurrence (adjusted OR 8.9), as was exposure to ACEI and ARBs with nephrotoxic agents (adjusted OR 2.7). Severity of cytokine release syndrome, and CAR-T–associated encephalopathy were associated with AKI in univariate analysis. Severe AKI was associated with reduced progression-free survival. **Conclusions**: Acute kidney injury commonly occurs following CAR-T cell therapy, and its occurrence was closely linked to pre-existing renal comorbidities and cumulative exposure to nephrotoxic agents and ACEI and ARBS. The severity of AKI was associated with reduced overall survival and progression-free survival.

## 1. Introduction

The introduction of chimeric antigen receptor T-cell (CAR-T) therapy over the past fifteen years has substantially improved the prognosis for various diseases, particularly hematologic malignancies. This new therapy induces remission in patients with refractory B-cell malignancies who have already undergone numerous treatments. In pivotal clinical trials conducted in patients with relapsed/refractory aggressive B-cell lymphoma, the highest complete response rates range from 39% to 54%, while one-year overall survival ranges from 40% to 45% and persists over time [1,2,3,4]. Its indications have gradually expanded to include relapsed/refractory acute lymphoblastic leukemia, chronic lymphocytic leukemia, mantle cell lymphoma, and multiple myeloma, with promising results [5,6,7,8,9].

However, the enthusiasm generated by these results must be tempered by the adverse effects specific to this treatment. The CAR’s high affinity for its antigen is the basis for its effectiveness in killing tumours, but it also leads to significant toxicity due to supraphysiological stimulation and the release of inflammatory cytokines [10,11,12]. This most often manifests as the onset of a cytokine release syndrome (CRS)—a systemic inflammatory reaction characterized by fever, hypotension, and capillary leak syndrome—and sometimes by the onset of ICANS or macrophage activation syndrome/hemophagocytic lymphohistiocytosis (HLH/MAS). As a result, organ dysfunction may occur, with acute kidney injury (AKI) appearing to be the most common [13,14,15,16,17,18,19].

The incidence of AKI following CAR-T therapy ranges from 5% to 34% depending on the study. It has been variably associated with higher mortality and prolonged stays in intensive care [17,18,19]. However, the data currently available remain limited and are based primarily on cohort studies with a small number of participants. In fact, there is still little detailed information available on AKI following CAR-T cells, its clinical course, and its prognosis.

In this study, we aimed to estimate, within a cohort of patients treated with CAR-T cells, the cumulative incidence of AKI, to characterize its clinical presentation, to explore the underlying pathophysiological mechanisms, and to clarify long-term outcomes, including renal recovery and the impact of kidney injury on progression-free survival. We also sought to identify risk factors for the development of AKI in these patients.

## 2. Patients and Methods

This study is a single-center, retrospective cohort study.

### 2.1. Patients

Chimeric antigen receptor T-cell therapy was introduced at our hospital in 2019. Since then, approximately 40 patients with hemopathy have received this treatment each year. The indications for this treatment are first determined by the hematology team and then reviewed by a multidisciplinary team that includes, among others, intensive care specialists. Treatment is initiated in the hematology department, where patients are closely monitored. If any of these patients develops a secondary or treatment-related complication that is sufficiently serious, they are admitted to the intensive care unit and followed simultaneously with the hematology team. Accordingly, patients with CRS or immune cell-associated neurotoxicity syndrome (ICANS) grade 2 or higher were promptly transferred to our medical ICU specialized in the management of immunocompromised patients. Patient health care directives are collected whenever CAR-T cell therapy is decided on. Our policy is that the decision to admit to ICU and to intensively treat this population should be made by both the intensivists and the haematologists and according to the patient and family wishes.

Consecutive adult (>18 years old) patients admitted to the hematology ward at the University Hospital of Montpellier (Montpellier, France) who received a CAR-T cell infusion therapy between 1 April 2019, and 3 December 2023, were included in this study. Among them, those admitted to the ICU were identified and monitored as well. Patients with stage IV and V chronic kidney disease (CKD) were excluded from our analysis because of their greater propensity to require RRT. Patients who objected to their data collection were also excluded from the study.

### 2.2. Data Collection

Clinical and laboratory data were collected retrospectively from patients during their stay in the hematology department and, where applicable, in the intensive care unit (ICU). Data were abstracted from electronic medical records. The laboratory data were electronically validated by clinical pathologists. Data extraction was performed without knowledge of the subsequent prognosis. Demographic characteristics, medical history, presenting symptoms and treatments were collected. Data were collected on the number of ACE inhibitors or ARBs and nephrotoxic agents (aminoglycosides, vancomycin, acyclovir.) administered prior to the onset of AKI. Laboratory values were recorded at admission and during patient follow-up. Estimated glomerular filtration rates (eGFR) were recorded before the patient admission to assess the existence and the stage of CKD and after hospital discharge to evaluate kidney function recovery.

### 2.3. CAR-T Cell Therapy

CAR T-cell quantification was performed on peripheral blood samples at the following post-infusion time points: days 3, 7, 14, 30, and 60. It was based on flow cytometry acquisition of a target of 50,000 total lymphocytes. Flow cytometry acquisition of CAR T-cell was carried out on a FACSCanto II flow cytometer (Becton Dickinson, Franklin Lakes, NJ, USA) and were analysed using FACS Diva NovoExpress software (Becton Dickinson, Franklin Lakes, NJ, USA). CAR expression was assessed within CD3-gated T-cells using the CD19 CAR detection reagent (Miltenyi Biotec, Bergisch Gladbach, Germany).

Absolute CAR T-cell counts were calculated using results from complete blood counts. When total leukocytes were below 0.5 × 10^9^/L, absolute quantification of CAR T cells was not available. Area under the curve (AUC) was calculated using the trapezoidal rule. Quantification was characterized by pharmacokinetic parameters: maximum concentration reached (C_max_), time to C_max_ (T_max_) and AUC values from day 3 to each follow-up time point [20,21].

### 2.4. Definitions

Baseline creatinine was obtained during patient follow-up, at least 3 months before CAR-T cell infusion. CKD was defined and staged according to the 2024 KDIGO guidelines based on GFR, hematuria and albuminuria categories [22,23].

AKI was defined and staged according to the 2012 KDIGO guidelines based on both serum creatinine and urine-output [24]. It was considered severe if it was KDIGO stage 2 or higher. The causes of this AKI were classified as septic, hypovolemic, secondary to tumor lysis syndrome, or other conventional causes. It was considered to be associated with CRS when the aforementioned causes were ruled out and the CRS was grade 2 or higher. Decisions regarding the initiation, discontinuation, and modalities of renal replacement therapy (RRT) were made by the physician in charge of the patient. Recovery of kidney function was defined as a reduction from the peak AKI stage based on KDIGO criteria at hospital discharge or the discontinuation of RRT. Recovery of kidney function was considered complete if RRT was discontinued and kidney function returned to baseline. Patients were considered to have “kidney sequelae” if they did not return to baseline kidney function or remained on RRT at hospital discharge.

The definitions and grades for CRS and ICANS were based on the classification of the American Society for Transplantation and Cellular Therapy (ASTCT) [25], with grades 3 and 4 both considered severe.

### 2.5. Outcomes

Our objective was to assess the incidence and severity of AKI occurring following CAR-T cell therapy, to analyse its progression and its impact on survival. Associated risk factors were also identified.

After hospital discharge, surviving patients were followed by the hematological team (every month in the first year, every three months in the second year, and then annually in subsequent years) for at least 3 years, and mortality at one and 3 years was assessed.

Overall survival (OS) was defined as the time elapsed from CAR-T cells infusion to the date of death from any cause. Progression-free survival (PFS) was defined as the time between CAR-T cells infusion to the date of the first hematological event: progression, relapse or all-cause death.

### 2.6. Statistical Analysis

Continuous data were described as mean and standard deviation or median and interquartile range (IQR) and categorical data are presented as numbers and percentages of total. Comparison of characteristics between patients with and without AKI was made using Student’s t-test and chi-square or Fisher’s exact test, as appropriate. Variables with *p* < 0.20 in univariate analysis, along with clinically relevant covariates, were included in multivariable logistic regression models. Given that 45 events occurred, 5 covariates were included in the model; namely age, stage of CRS, chronic kidney disease, the number of ACEI/ARBs and nephrotoxics, and use of mechanical ventilation. Kaplan–Meier survival up to 60 months after CAR-T cell infusion were stratified according to AKI KDIGO staging and compared using a log-rank test after controlling the proportional hazard hypothesis. *p* values < 0.05 were considered statistically significant, and all statistical tests were two-sided. Statistical analyses were performed using R version 3.4.2 (R Foundation for Statistical Computing, Vienna, Austria; https://www.R-project.org/).

## 3. Results

### 3.1. Patient Baseline Characteristics

**Figure 1:**
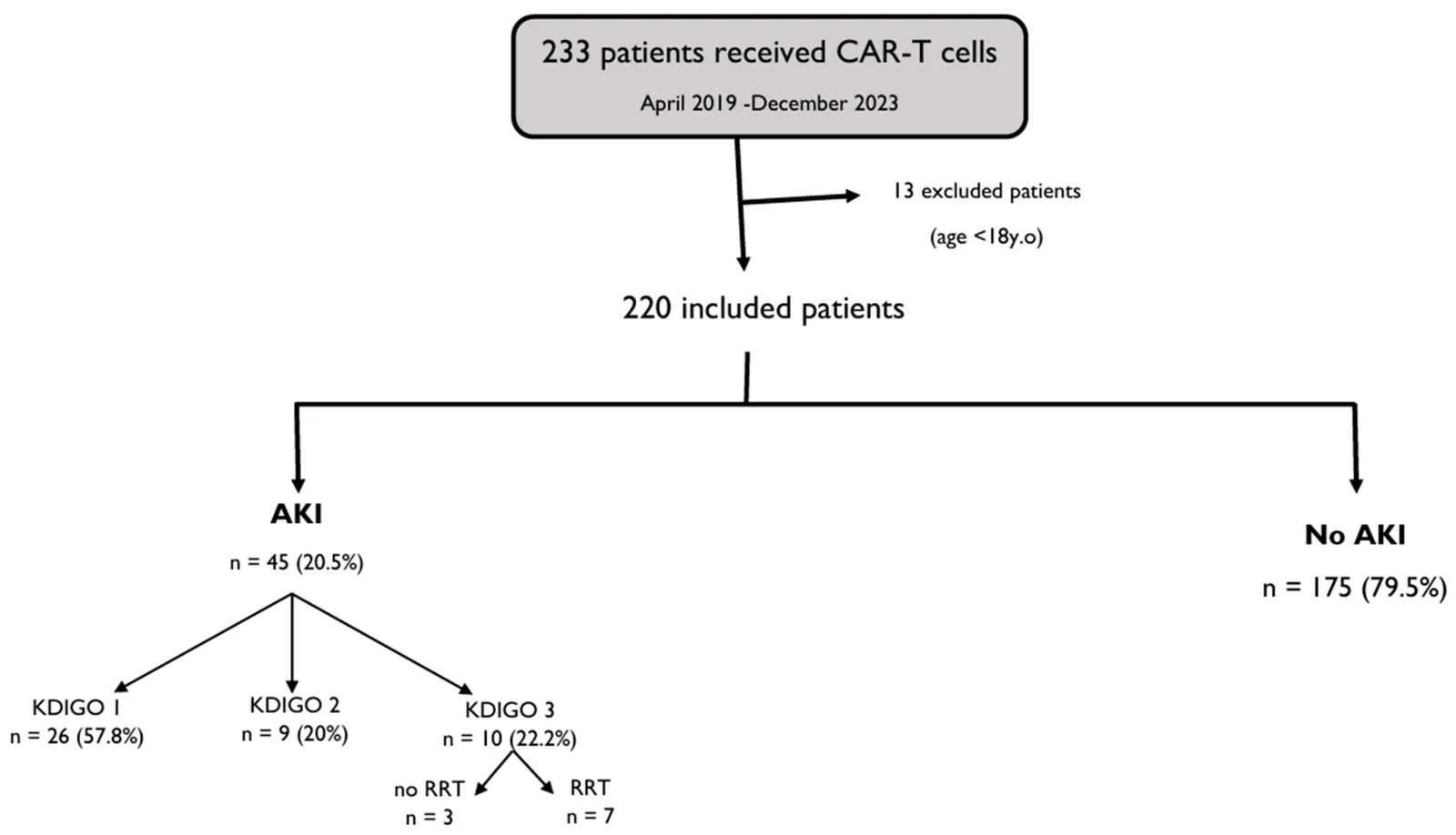
Flow chart of the studied population. CAR-T: Chimeric Antigen Receptor-T, AKI: Acute Kidney Injury, RRT: Renal replacement therapy, KDIGO: Kidney Disease Improval Global Outcome.

Of the 233 patients who received CAR-T cell therapy during the study period, 220 patients were clinically monitored and included in the study. Only 13 patients were excluded because they were under 18 years of age (Figure 1). The patient characteristics at the time of CAR-T cell infusion are presented in Table 1. The study population was predominantly male (men: 65%) and had a mean age of 63 ± 11.5 years. Hypertension was present in 60 patients (27.3%) and diabetes mellitus in 23 patients (10.5%). Chronic kidney disease (CKD), primarily nephroangiosclerosis, was also observed in 57 patients (25.9%), of whom 45 (20.5%) were at stage 2 and 12 (5.5%) at stage 3A. ACEI or ARBs were administered to 15% (34 out of 220) of the patients prior to treatment. More than 80% of patients (177 out of 220) had a performance status scale score of less than 2.

The underlying hematologic malignancy was mainly a B-cell lymphoma, primarily diffuse large B-cell lymphoma: 187 (85%). CAR-T cell therapy was preceded by a median of 2 (IQR: 2–3.25) lines of chemotherapy. The type of autologous CAR-T cells administered is specified in Table 1.

During the 4 weeks prior to the administration of CAR-T cells, infectious complications occurred in 27 (12.3%) patients, and tumor-related complications in 12 (5.5%) patients. Additionally, 19 (8.6%) patients received steroids during this period. Treatment had to be delayed in 17 (7.7%) patients.

### 3.2. Patient Outcomes Following CAR-T Therapy

The in-situ expansion of CAR-T cells was quantified from day 3 through day 60 after injection, showing a gradual and significant increase (Table 2). Cytokine release syndrome occurred in 200 patients (90.9%), lasting a median of 7 days; the majority of these cases was Grade 1 (129/200, 64.5%). ICANS, with a median duration of 12 days, was observed in 69 patients (31.4%), nearly half of whom (30/69; 43.4%) had a severe form (Grade 3 or 4). An Hemophagocytic lymphohistiocytosis (HLH) was diagnosed in 2 patients. Most patients (93.4%) were administered antibiotics. All these data are summarized in Table 2.

Of the 220 patients studied, 53 (24%) were admitted to the ICU. Their demographic characteristics were similar to those of the general population. The primary reason for admission was a CRS, with or without an ICANS (29/53; 54.7%). At ICU admission, severity scores were 50 ± 18 for SAPS II and 4 ± 3 for SOFA. During their stay in ICU, a CRS was observed in 52 patients (98.1%), an ICANS in 37 (69.8%) of them; vasopressors were administered to 20 patients (37.7%) for a median duration of 3 [1–5.2] days and mechanical ventilation was used in 12 patients (22.6%) for a median duration of 5 [2.7–8] days. Median ICU length of stay was at 4 [3–8] days. Five patients (5 out of 53) died in the ICU, bringing the ICU mortality rate to 9.4%.

### 3.3. Acute Kidney Injury Cumulative Incidence, Clinical Presentation and Outcome

Over the study period, 45 out of 220 patients (20.5%) experienced AKI with a mean onset of 4 ± 3 days after CAR-T cell infusion. Of these, 26 (57.8%) were classified as KDIGO stage 1, 9 (20%) as stage 2, and 10 (22.2%) as stage 3 (Table 3). Of these patients, 24 out of 45 were treated in the ICU, 10 of whom had developed AKI prior to admission.

AKI was primarily multifactorial in origin and associated with CRS. Most of AKI patients (24 out of 45) were previously treated by ACEIs or ARBs. In many cases (27/45, 60%) exposure to nephrotoxic agents—mainly aminoglycosides—has been observed prior to the onset of AKI. The median number of nephrotoxic agents and ACEIs or ARBs administered was at 1 (0–2). Seven patients required RRT for a median duration of 11 [3.5–23.5] days. All 5 patients who died had severe AKI and were admitted to the ICU (5/45; hospital mortality: 11.1%). These results are displayed in Table 3 and Table 4.

### 3.4. Risk Factors of AKI

We compared patients with AKI to those without it (Table 4). Univariate analysis shows that pre-existing hypertension (*p* = 0.01) and chronic kidney disease (*p* = 0.001) were significantly associated with the occurrence of AKI, as was primary kidney damage due to a malignant hematologic disorder (*p* = 0.006). CAR-T cell expansion was significantly greater in the AKI group, as evidenced by higher AUC (days 7 to 30) and Cmax values (*p* = 0.001). The occurrence of CRS and/or ICANS, particularly in their severe forms, was significantly associated with that of AKI. In fact, the use of targeted therapies for CRS and/or ICANS- tocilizumab (*p* = 0.018), dexamethasone (*p* < 0.001) et anakinra (*p* < 0.001)- was significantly more common in the AKI group. In addition, peak plasma levels of CRP (*p* < 0.001) and ferritin (*p* = 0.002) were significantly higher in patients with AKI. A higher number of nephrotoxic agents and ACEI or ARBs was also associated with the occurrence of AKI. Among patients admitted to the ICU, those with AKI had significantly higher SAPS II and SOFA scores, required vasopressors and mechanical ventilation more frequently, and had a higher mortality rate (21% vs. 0%).

**Figure 2:**
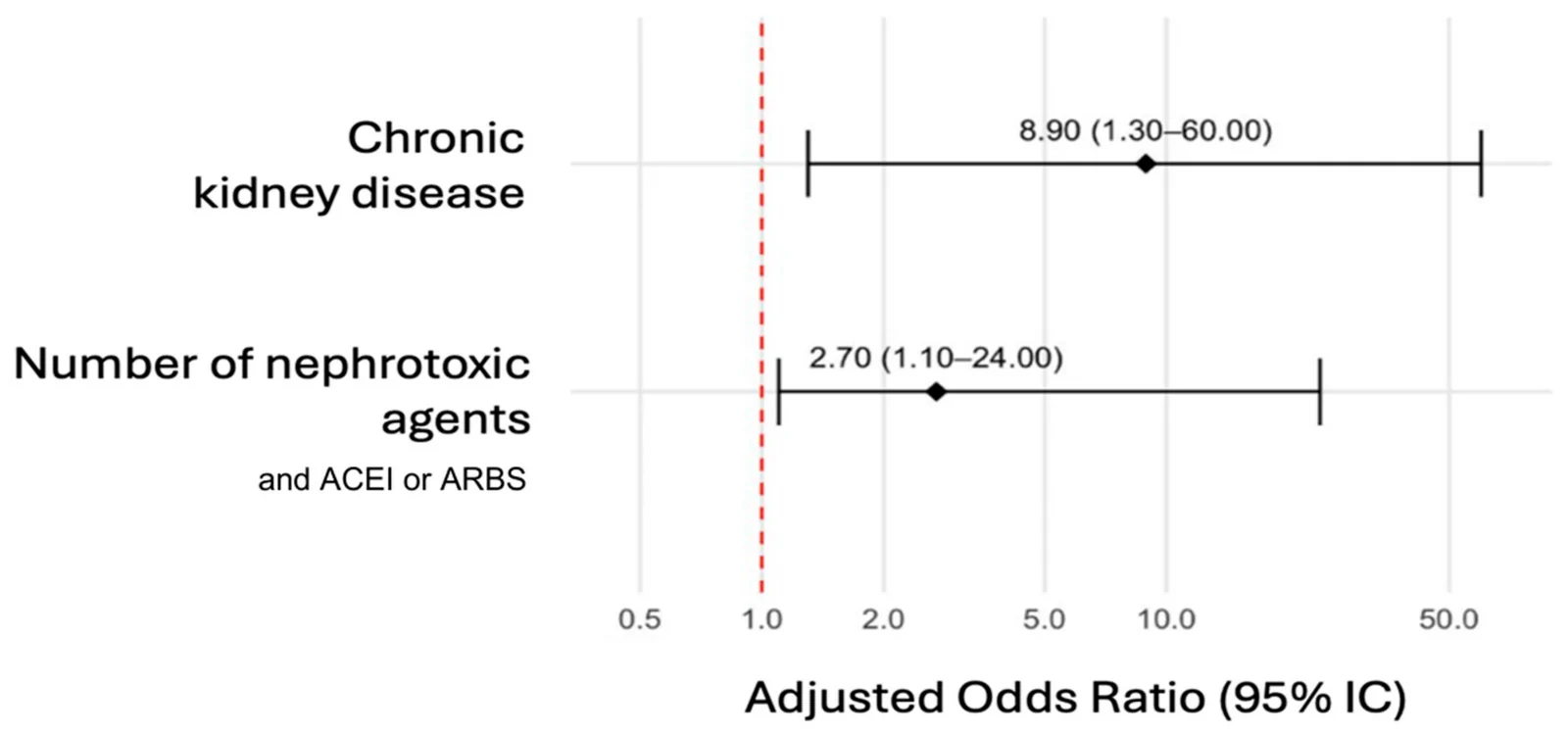
Multivariable analysis showing significant factors associated with AKI. AKI: Acute Kidney Injury.

However, the multivariate analysis identified only two factors independently associated with an increased risk of AKI: pre-existing CKD (adjusted OR = 8.9, 95% CI: 1.3–60.0, *p* = 0.02) and number of nephrotoxic agents and ACEI or ARBs (aOR = 2.7, 95% CI: 1.1–24.0, *p* = 0.04) (Figure 2).

**Figure 3:**
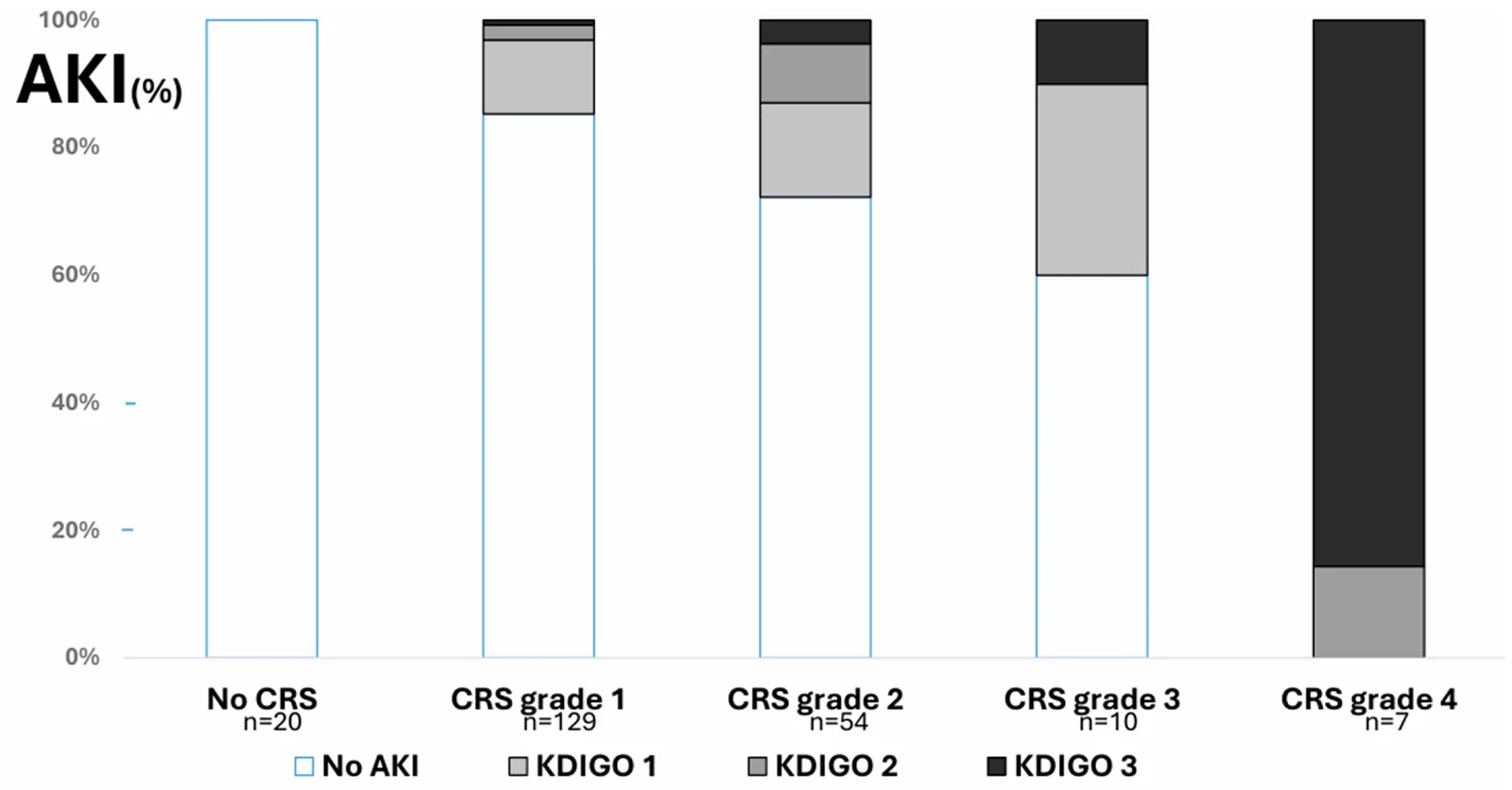
Distribution of patients without and with AKI at different KDIGO stages according to the severity of CRS. AKI: Acute Kidney Injury, CRS: Cytokine Release Syndrome.

Figure 3 shows the percentage of patients who did not develop or did develop AKI at the various KDIGO stages, based on the severity of CRS. A significant proportion of patients with a CRS score of 2 or higher (15 out of 71 patients, 21%) had severe AKI—that is, stage 2 or 3 according to the KDIGO classification—while among the 149 patients with a CRS score of 0 or 1, only 4 (2.7%) had severe AKI (significant difference, *p* = 0.016). In addition, the occurrence of severe AKI was significantly associated with a high ICANS score (*p* = 0.024).

### 3.5. Management and Monitoring of Renal Function in Patients with AKI

Of the 45 patients with AKI, 7 (15.6%) received RRT for a median duration of 11 days (IQR: 3.5–23.5); 5 of these patients died during their stay in the ICU, while the 2 survivors progressed to end-stage CKD requiring chronic haemodialysis. Of the 38 surviving patients who did not receive RRT, 31 recovered their previous renal function, including 19 who had pre-existing CKD (Table 3).

### 3.6. Overall Survival and Progression-Free Survival in Patients

The median overall survival was 48 months (IQR: 9–not reached). It was significantly shorter in patients with severe AKI compared with those with mild AKI or no AKI (log-rank *p* = 0.011).

**Figure 4:**
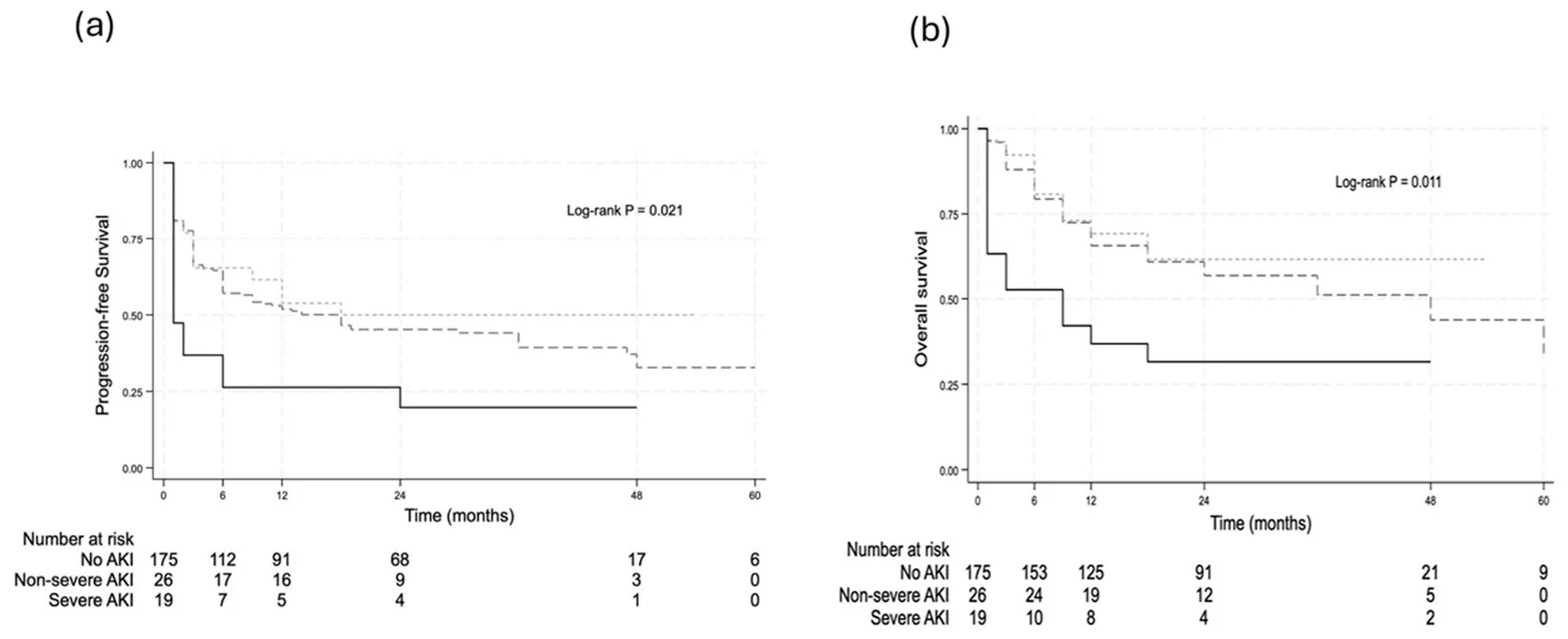
Kaplan-Meier survival analysis of progression-free (**a**) and overall (**b**) survival according to AKI staging. AKI: Acute Kidney Injury; No AKI (dark grey), AKI KDIGO stage 1 (light grey), and severe AKI KDIGO stage 2 and 3 (black).

The median progression-free survival was 12 months (IQR: 2–not reached). It was 1 month (IQR: 1–24) for patients with severe AKI, compared with 18 months (IQR: 3–not reached) for patients with no AKI or mild AKI (log-rank *p* = 0.021). The corresponding Kaplan-Meier curves, illustrating the association between the severity of AKI and mortality, are shown in Figure 4.

## 4. Discussion

In this single-centre cohort of 220 patients treated with CAR-T cell therapy for hematologic malignancies, AKI occurred in approximately 20% of patients, with severe AKI (KDIGO stage ≥ 2) occurring in 8.6% of them. The onset of AKI in this context was an indicator of disease severity, particularly when it was severe and required RRT. Pre-existing CKD, previous treatment with ACEI or ARBs and exposure to nephrotoxic agents were significantly associated with an increased risk of AKI. Overall survival and progression-free survival decreased as the KDIGO stage of AKI was ≥2.

The overall incidence of AKI at 20% that we observed is comparable to that reported in previous studies, with incidences ranging from 5% to 30% [15,16,17,18]. A systematic review of 22 cohort studies estimated an overall incidence of AKI in adult patients treated with CAR-T therapies of 24.1% [18]. A higher incidence, at 34%, was reported among the subgroup of patients admitted to ICU [19], whereas it was 45% (24/53) in our study. However, the severity of our ICU patients’ condition appears to be much greater.

Post CAR-T cell AKI is a complex and likely multifactorial condition. Various pathophysiological mechanisms and hypotheses have been proposed by several authors to explain its onset. AKI can be directly related to CRS, mainly through hypotension and vasodilatory shock, which reduce renal perfusion and may progress from prerenal injury to ischemic acute tubular necrosis [26,27]. It is the elevation of cytokine levels, particularly interleukin-6 (IL-6) and interferon-γ (IFN-γ), that is thought to cause this vasodilatory shock, accompanied by capillary leakage, third-spacing, and direct renal inflammation. In our study population, more than 90% of patients presented with CRS, primarily grade 1 (64.5%). However, all patients with AKI had CRS, and it was more severe (grade ≥ 2) than in those without AKI. The more severe the CRS, the higher the incidence of AKI, as previously reported [15,16,17,18,19]. The most severe cases of CRS do indeed exhibit higher peaks of IL-6, IL-10, and IFN-γ, as well as frequent hyperthermia, accompanied by dehydration and a decrease in effective blood volume, making them more susceptible to developing AKI [13,17,19,28]. A mechanism involving direct renal toxicity from CAR-T cells has also been suggested, although it is rarer. This mechanism is thought to involve both cytokine overproduction and renal infiltration by T cells [29,30]. The so-called “on-target-off-tumor” phenomenon is based on the possibility that CAR-T cells may attack both tumor cells and kidney cells due to shared antigens such as CD19, which is widely expressed on both healthy and malignant B cells [31]. The quantification over time of T-cell expansion in our patients revealed by univariate analysis that it was significantly greater in patients with AKI. However, the difference is only significant starting at D7, by which time the AKI—when it occurred—was already established. It remains to be determined whether the expansion of CAR-T cells could worsen AKI. Tumor lysis syndrome can rarely occur and may also lead to AKI [3,7,32]. It was identified in only one patient in our cohort. Lastly, sepsis is one of the main leading causes of AKI, as CAR-T cell recipients are particularly susceptible to it. Sepsis-related AKI results from renal hypoperfusion and inflammation leading to acute ischemic tubular necrosis [28]. In fact, sepsis was identified as the primary cause of AKI in nearly a quarter of our patients with AKI. However, the coexistence of multiple conditions can complicate the identification of the underlying cause of renal failure.

Our objective was to identify risk factors associated with the onset of AKI. Our results showed that pre-existing CKD was an independent risk factor for AKI. These findings contrast with those of Wood et al., who reported that prior renal impairment was not associated with an increased risk of AKI [33], and with those of Vincendeau et al., who found no association between prior CKD and AKI in ICU patients [19]. However, these two studies included a smaller proportion of patients with CKD in their cohorts (10% and 4%, respectively), compared with 26% in our overall cohort. Furthermore, Farrooqui et al. [34], in an evaluation of 83 patients treated with CAR-T therapy, observed that AKI occurred in 1 out of every 6 patients and that pre-existing impaired kidney function was a predictor of the risk of its onset. The second risk factor for AKI that we identified was prior treatment with ACEI or ARBs and exposure to nephrotoxic agents. These patients are often treated with nephrotoxic agents such as aminoglycosides, vancomycin, or iodinated contrast media. They may also be exposed to an overdose of chronically administered medications, particularly ACE inhibitors or ARBs, which can further compromise renal function as the estimated GFR declines. We observed that the accumulation of multiple nephrotoxic agents was even more strongly associated with the onset of AKI, as reported previously [33].

Our findings show a significant survival rate within this cohort, bearing in mind that these results may be influenced by the highly selective nature of the study population [7]. However, AKI has had an impact on both short- and long-term outcomes, particularly in severe cases. The association between severe AKI (KDIGO stages 2–3) and mortality has been consistently demonstrated in previous studies. Wood et al. showed that AKI was associated with reduced overall survival and progression-free survival, and that patients with AKI had a higher rate of admission to the ICU [33]. In their cohort of 78 CAR-T cell recipients, Gupta et al. observed higher 60-day mortality among the 15 patients who developed AKI [17]. In ICU settings, Vincendeau et al. further demonstrated that KDIGO stage 2 or higher AKI remained an independent predictor of in-hospital mortality in a multivariate analysis [19]. Our results are consistent with these observations, although the study was not specifically designed to identify factors associated with mortality. One of the key findings of our study is that most patients who survived AKI recovered their previous kidney function. In fact, only 22% (9/40) of survivors developed new renal sequelae (stage 2 to 4 CKD), and two of them progressed to end-stage CKD (one without pre-existing CKD and the other with stage 2 CKD prior to treatment). Nevertheless, close clinical and laboratory monitoring, as well as long-term specialized nephrological follow-up, must be recommended [14].

Several limitations of this study should be acknowledged. Due to its single-centre nature, its results should be generalized with caution, as patient characteristics, treatment protocols, and institutional practices vary from one medical centre to another. However, institutional practices follow current guidelines for the management of CRS and ICANS. Furthermore, this introduces biases inherent in data collection and analysis. Finally, the relatively small number of AKI cases likely limited statistical power and may have prevented the detection of other associations identified in the univariate analysis. However, this study has the largest number of patients to provide a comprehensive analysis of AKI following CAR-T cell therapy.

## 5. Conclusions

The occurrence of AKI following CAR-T therapy was relatively common in our cohort. In its severe form, it increased mortality. It was significantly associated with pre-existing CKD. Furthermore, the accumulation of ACEIs or ARBS and nephrotoxic agents was a contributing factor. Due to its close association with CRS, effective prevention and prompt management are essential to mitigate renal toxicity. Future studies should aim to elucidate the underlying pathophysiological mechanisms and identify predictive factors for AKI, including potential biomarkers.

## Figures and Tables

**Table 1:** Characteristics of the study population.

	Total Population n = 220
**Age**, year [mean (SD)]	63 (11.5)
**Male sex**, n (%)	143 (65%)
**Pre-existing conditions**, n (%)	
Hypertension	60 (27.3)
CKD	57 (25.9)
Congestive heart failure	49 (22.3)
Diabetes mellitus	23 (10.5)
Chronic liver disease	19 (8.6)
Amyloidosis	1 (0.5)
**ECOG performance status scale**, n (%)	
0	55 (25)
1	122 (55.5)
2	31 (14.1)
3	10 (4.5)
4	2 (0.9)
**Hematological disease**, n (%)	
B cell lymphoma	187 (85)
Multiple myeloma	29 (13.2)
Acute lymphoblastic leukemia	4 (1.8)
**Number of previous therapeutic lines** [median (IQR)]	2 (2–3.25)
**CAR-T cell**, n (%)	
Axicabtagene ciloleucel (Yescarta™)	134 (60.9)
Idecabtagene vicleucel (Abecma™)	39 (17.7)
Tisagenlecleucel (Kymriah™)	25 (11.4)
Brexucabtagene autoleucel (Tecartus™)	16 (7.3)
Lisocabtagene maraleucel (Breyanzi™)	6 (2.7)

CKD = Chronic Kidney Disease, ECOG = Eastern Cooperative Oncology Group, CAR = Chimeric Antigen Receptor.

**Table 2:** Patient outcomes following CAR-T cells therapy.

	Total Population n = 220
**CAR-T cell expansion**	
**AUC**, G/L D3	4.02 (9.10)
D60 *	2034 (2966)
**C_max_**, G/L [mean (SD)]	220 (357)
**T_max_**, days [mean (SD)]	11.3 (6.2)
**CRS**, n (%)	200 (90.9)
**CRS duration**, day [median (IQR)]	7 (5–10)
1	129 (64.5)
2	54 (27)
3	10 (5)
4	7 (3.5)
**ICANS**, n (%)	69 (31.4)
**Severe ICANS**, n (%)	30 (13.6)
**ICANS duration**, day [median (IQR)]	12 (9–14)
Use of **Tocilizumab**, n (%)	93 (42.3)
Use of **Dexamethasone**, n (%)	74 (33.6)
Steroids boluses, n (%)	9 (4.1)
Use of **Anakinra**, n (%)	23 (10.5)
**Peak CRP**, mg/L [mean (SD)]	131 (103)
**Peak Ferritin**, µg/L [mean (SD)]	3406 (11,890)
Use of **GCS-F**, n (%)	147 (66.8)
**HLH**, n (%)	2 (0.9)
**Antibiotics exposure**, n (%)	205 (93.4)
**In Hospital Mortality**, n (%)	12 (5.5)

ICU = Intensive Care Unit, CRS = Cytokine Release Syndrome, ICANS = Immune effector Cell-Associated Neurotoxicity Syndrome, CRP = C-Reactive Protein, HLH = Hemophagocytic LymphoHistiocytosis. * data from 208 patients, 12 of whom died before the 60th day.

**Table 3:** Acute kidney injury: incidence, clinical presentation and follow-up.

	Total Population n = 220
**AKI**, n (%)	45 (20.5)
**AKI main etiology**, n (%)	
CAR-T	28 (62.2)
Sepsis/Hypovolemia	11 (24.4)
Tumoral lysis syndrome	1 (2.2)
Other	5 (11.2)
**AKI delay from CAR-T cell infusion**, days [mean ± sd]	**4 ± 3**
**AKI stage,** n (%)	
1	26 (57.8)
2	9 (20)
3	10 (22.2)
**AKI duration**, days [median (IQR)]	4 (3–9)
**Number of nephrotoxic agents and ACEI/ARBs** [median (IQR)]	1 (0–2)
**RRT**	7 (3.2)
**RRT duration**, days [median (IQR)]	11 (3.5–23.5)
**In-Hospital mortality, n(%)**	5 (11.1)
**CKD post-AKI at hospital discharge**, n (%)	9 (20)
<4	7 (15.5)
5	2 (4.4)

CAR = Chimeric Antigen Receptor, AKI = Acute Kidney Injury, RRT = Renal Replacement Therapy, CKD = Chronic Kidney Disease, ACEI = Angiotensin-Converting Enzyme Inhibitors, ARB = Angiotensin II Receptor Blockers.

**Table 4:** Univariate analysis comparing patients without and with AKI.

	No AKI 175	AKI 45	*p*
**Age**, year [mean (SD)]	62 (12)	67 (10)	0.07
**Male** sex, n (%)	111 (63.4)	32 (71.1)	0.337
**Pre-existing conditions**, n (%)			
Hypertension	41 (23.4)	19 (42.2)	**0.011**
Diabetes mellitus	19 (10.9)	4 (8.9)	0.702
CKD	37 (21.1)	20 (44.4)	**0.001**
**Hematological disease**			0.735
B cell lymphoma	149 (85.1)	38 (84.4)	
Multiple myeloma	22 (12.6)	7 (15.6)	
Acute lymphoblastic leukemia	4 (2.3)	0	
**Kidney involvement**	1 (0.6)	3 (6.7)	**0.006**
**Number of previous therapeutic lines** [median (IQR)]	2 (2–4)	2 (2–3)	0.259
**CAR-T cell expansion**			
**AUC**, G/L × days			
D3	4.3 (9.6)	2.8 (6.7)	0.463
D7	187 (286)	684 (1052)	**<0.001**
D14	806 (979)	1937 (2461)	**0.001**
D30	1458 (1929)	2704 (3143)	**0.025**
D60	1841 (2783)	2950 (3683)	0.175
**C_max_**, G/L [mean (SD)]	155 (218)	458 (601)	**<0.001**
**T_max_**, days [mean (SD)]	11.4 (6.3)	11 (5.8)	0.742
**Complications after CAR-T cells infusion**			
**CRS**	155 (88.6)	45 (100)	**0.017**
CRS **duration**, days [median (IQR)]	7 (5–10)	8 (6–10)	0.635
CRS **maximum grade**			**<0.001**
1	110 (62.3)	19 (42.2)	
2	39 (22.3)	15 (33.3)	
3	6 (3.4)	4 (8.9)	
4		7 (15.6)	
**ICANS**, n (%)	47 (26.9)	22 (48.9)	**0.004**
Severe ICANS, n (%)	14 (8)	16 (35.6)	**<0.001**
ICANS **duration**, days [median (IQR)]	12 (9–15)	11 (11–14)	0.068
**Tocilizumab**, n (%)	67 (38.3)	26 (57.8)	**0.018**
**Anakinra**, n (%)	12 (6.9)	11 (24.4)	**<0.001**
**Dexamethasone**, n (%)	46 (26.3)	28 (62.2)	**<0.001**
Steroids boluses, n (%)	2 (1.1)	7 (15.6)	**<0.001**
Peak **CRP**, mg/L [mean (SD)]	111 (87)	210 (123)	**<0.001**
Peak **Ferritin**, µg/L [mean (SD)]	2100 (3265)	8368 (24,855)	**0.002**
**Nephrotoxic agents exposure**, n (%)	66 (37.7)	27 (60)	**0.007**
**ACEI or ARBS,** n (%)	10 (5.7)	24 (53.3)	**<0.001**
**Nephrotoxic agents** and **ACEI/ARBS n,** [median (IQR)]	0 (0–1)	1 (0–2)	**<0.001**
**In-Hospital mortality**, n (%)	7 (4)	5 (11.1)	**0.010**

SAPS II = Simplified Acute Physiology Score, AKI = Acute Kidney Injury, CKD = Chronic Kidney Disease, CAR = Chimeric Antigen Receptor, LDH = Lactate DesHydrogenase enzyme, AUC = Area Under the Curve, CRS = Cytokine Release Syndrome, ICANS = Immune effector Cell-Associated Neurotoxicity Syndrome, ACEI = Angiotensin-Converting Enzyme Inhibitors, ARB = Angiotensin II Receptor Blockers.

## Data Availability

Data is available from the corresponding author upon reasonable request.

## List of Abbreviations

**ACEI**	Angiotensin-converting enzyme inhibitor
**AKI**	Acute kidney injury
**ARB**	Angiotensin II receptor blocker
**ASTCT**	American society for transplantation and cellular therapy
**AUC**	Area under the curve
**CAR-T**	Chimeric antigen receptor T-cell
**CRP**	C-reactive protein
**CRS**	cytokine release syndrome
**CKD**	Chronic kidney disease
**ECOG**	Eastern cooperative oncology group, eGFR
**Estimated**	glomerular filtration rate
**G-CSF**	Granulocyte colony-stimulating factor
**HLH**	Hemophagocytic lymphohistiocytosis
**ICANS**	Immune cell-associated neurotoxicity syndrome
**ICU**	Intensive care unit
**IFN-γ**	Interferon-γ, IL-6 Interleukin-6
**IQR**	Interquartile range
**KDIGO**	Kidney disease improving global outcomes
**LDH**	Lactate dehydrogenase
**MAS**	Macrophage activation syndrome
**OR**	Odd ratio
**PFS**	Progression-free survival
**RRT**	Renal replacement therapy
**SAPSII**	Simplified acute physiology score II
